# Antibacterial polysaccharide-based hydrogel dressing containing plant essential oil for burn wound healing

**DOI:** 10.1093/burnst/tkab041

**Published:** 2021-12-22

**Authors:** Huanhuan Wang, Yang Liu, Kun Cai, Bin Zhang, Shijie Tang, Wancong Zhang, Wenhua Liu

**Affiliations:** Department of Biology & Guangdong Provincial Key Laboratory of Marine Biotechnology, Institute of Marine Sciences, College of Science, Shantou University, Shantou, Guangdong, 515063, P.R. China; Department of Biology & Guangdong Provincial Key Laboratory of Marine Biotechnology, Institute of Marine Sciences, College of Science, Shantou University, Shantou, Guangdong, 515063, P.R. China; Department of Biology & Guangdong Provincial Key Laboratory of Marine Biotechnology, Institute of Marine Sciences, College of Science, Shantou University, Shantou, Guangdong, 515063, P.R. China; Department of Biology & Guangdong Provincial Key Laboratory of Marine Biotechnology, Institute of Marine Sciences, College of Science, Shantou University, Shantou, Guangdong, 515063, P.R. China; Department of Plastic Surgery and Burn Center, Second Affiliated Hospital, Plastic Surgery Institute of Shantou University Medical College, Shantou, Guangdong, 515063, P.R. China; Department of Plastic Surgery and Burn Center, Second Affiliated Hospital, Plastic Surgery Institute of Shantou University Medical College, Shantou, Guangdong, 515063, P.R. China; Department of Biology & Guangdong Provincial Key Laboratory of Marine Biotechnology, Institute of Marine Sciences, College of Science, Shantou University, Shantou, Guangdong, 515063, P.R. China

**Keywords:** Carboxymethyl chitosan, Eucalyptus essential oil, Hydrogel, Antibacterial activity, Burn, Wound healing

## Abstract

**Background:**

Polysaccharide-based hydrogels have been developed for many years to treat burn wounds. Essential oils extracted from aromatic plants generally exhibit superior biological activity, especially antibacterial properties. Studies have shown that antibacterial hydrogels mixed with essential oils have great potential for burn wound healing. This study aimed to develop an antibacterial polysaccharide-based hydrogel with essential oil for burn skin repair.

**Methods:**

Eucalyptus essential oil (EEO), ginger essential oil (GEO) and cumin essential oil (CEO) were employed for the preparation of effective antibacterial hydrogels physically crosslinked by carboxymethyl chitosan (CMC) and carbomer 940 (CBM). Composite hydrogels were prepared and characterized using antimicrobial activity studies, Fourier-transform infrared spectroscopy, X-ray diffraction, scanning electron microscopy, gas chromatography-mass spectrometery, rheological analysis, viscosity, swelling, water loss rate and water vapor transmission rate studies. In addition, the biocompatibility of hydrogels was evaluated *in vivo* by cytotoxicity and cell migration assays and the burn healing ability of hydrogels was tested *in vivo* using burn-induced wounds in mice.

**Results:**

The different essential oils exhibited different mixing abilities with the hydrogel matrix (CMC and CBM), which caused varying levels of reduction in essential oil hydrogel viscosity, swelling and water vapor transmission. Among the developed hydrogels, the CBM/CMC/EEO hydrogel exhibited optimal antibacterial activities of 46.26 ± 2.22% and 63.05 ± 0.99% against *Staphylococcus aureus* and *Escherichia coli*, respectively, along with cell viability (>92.37%) and migration activity. Furthermore, the CBM/CMC/EEO hydrogel accelerated wound healing in mouse burn models by promoting the recovery of dermis and epidermis as observed using a hematoxylin–eosin and Masson’s trichrome staining assay. The findings from an enzyme-linked immunosorbent assay demonstrated that the CBM/CMC/EEO hydrogel could repair wounds through interleukin-6 and tumor necrosis factor-α downregulation and transforming growth factor-β, vascular endothelial growth factor (VEGF) and epidermal growth factor upregulation.

**Conclusions:**

This study successfully prepared a porous CBM/CMC/EEO hydrogel with high antibacterial activity, favorable swelling, optimal rheological properties, superior water retention and water vapor transmission performance and a significant effect on skin repair *in vitro* and *in vivo*. The results indicate that the CBM/CMC/EEO hydrogel has the potential for use as a promising burn dressing material for skin burn repair.

Highlights

- CBM/CMC hydrogels were prepared with essential oils by physical crosslinking, and CBM/CMC/EEO hydrogel characterization was extensively determined.
- CBM/CMC/EEO hydrogel displayed antimicrobial and cell migration activity.
- CBM/CMC/EEO hydrogel was proved to have burn wound healing activities.

## Background

Polysaccharide-based hydrogels have been developed for many years to treat burn wounds [1–5]. Burn and scald injuries of skin are ailments with high morbidity and mortality, affecting ~11 million people worldwide each year [6]. Burn and scald injuries damage the barrier function of the skin, thus leading to the loss of water, electrolytes and proteins from wounds. During the healing process, improper nursing care, like traditional bandages, cotton pads and ointments, generally delay wound healing as microorganisms invade the wounds to cause infection. Further, the neonatal granulation tissue easily adheres to the dressing, leading to secondary injury during dressing removal [7,8]. Compared to traditional materials, application of polysaccharide-based hydrogel dressings on wounds presents advantages, such as superior exudate absorbance, transparent material allowing the monitoring of healing, temporary and prompt wound coverage, wearing comfort, immediate relief from pain and minimization of possible damage due to the 3D and hydrophilic polymeric network of hydrogels [9].

To date, many macromolecular polysaccharides, such as starch, alginate, cellulose, chitoson, hyaluronic acid, carboxymethyl chitosan (CMC, a water soluble derivative of chitosan), etc., have been widely used to develop polysaccharide-based hydrogels. The use of polysaccharides as the primary hydrogel matrix to improve the hydrophilic performance of polymeric hydrogels has emerged as a significant research field [10–14]. In addition, the majority of polysaccharide-based hydrogels are usually comprised of additional cross-linked substances, owing to their poor mechanical properties and insufficient biological activity. For instance, in order to improve the biological activity and physical properties of polysaccharide hydrogels, a carbomer940 (CBM)/CMC /bletilla striata polysaccharide cross-linked hydrogel was reported in our previous study [15]. However, the hydrogel demonstrated poor antibacterial activity, which limited its application in the biomedical field.

A number of essential oils are frequently incorporated into polysaccharide-based hydrogels as active ingredients [16–18]. Essential oils extracted from aromatic plants contain numerous low molecular weight molecules derived from isoprene, including terpenoids and related terpenes, which generally exhibit superior biological activity. Essential oils have been reported to be active against gram-positive and gram-negative bacteria, along with having active skin tissue repair and antioxidation functions [18,19]. However, the direct application of essential oils is limited because they are unstable and fragile volatile compounds. Essential oils are easily degraded under various physico-chemical conditions, such as light, heat, oxidation, etc. [20]. In order to maintain their biological activity over a long period of time, along with reducing their volatility, increasing their effective utilization rate and providing controlled release [21], essential oils are generally encapsulated in hydrogel matrices, leading to their sustained release. Boccalon *et al*. reported that hydrogels prepared by incorporating polyvinyl alcohol (PVA), sodium alginate and borax displayed superior antimicrobial properties after loading with essential oil [16]. Moradi *et al*. also reported optimal antibacterial activity of chitosan/PVA/thyme oil hydrogels against *Escherichia. coli* (*E. coli*) and *Staphylococcus aureus* (*S. aureus*) [17]. Therefore, the addition of essential oils to polysaccharide-based hydrogel matrices, rather than using them directly on wounds, can be instrumental in enhancing their physical stability and decreasing their volatility, thus leading to superior biological activity of the polysaccharide hydrogels.

In summary, polysaccharide hydrogels with improved antibacterial activity have been developed in this study. Natural hydrogel dressings for the treatment of burn wounds were subsequently prepared by physical crosslinking of CBM, CMC and plant essential oils (eucalyptus essential oil (EEO), ginger essential oil (GEO) and cumin essential oil (CEO)). The dressings were prepared by employing the optimal proportion of raw materials to achieve superior antibacterial performance. The morphology, rheological behavior, swelling, water loss and water vapor transmission properties of the hydrogels were systematically investigated. In addition, the burn wound healing of the composite hydrogel dressing was evaluated through *in vivo* and *in vitro* animal experiments.

## Methods

### Materials

EEO, GEO and CEO were purchased from ZRZR Biotechnology Co. Ltd, Guangdong, China. CMC (molecular weight concentrated at 195.7 kDa and 2.0 kDa; the degree of substitution was 73.73% (Figures S1–S5, see online supplementary material); and CBM (molecular weight concentrated at 1894.7 kDa and 15.8 kDa; CAS:54182–57-9) were supplied by Yuanye biotechnology Co. Ltd, Shanghai, China. Triethanolamine was procured from Aladdin Reagent Co. Ltd, Shanghai, China. Luria–Bertani (LB) broth and the L-929 cell migration and cytotoxicity detection kit were supplied by Solarbio (Beijing, China). 3-(4,5-Dimethyl-2-thiazolyl)-2,5-diphenyl-2-H-tetrazolium bromide (MTT) was purchased from Sigma (Shanghai, China). Chloral hydrate was obtained from Shanghai Macklin Biochemical Co. Ltd, China. The reagents used in the study were of analytical grade.

### Preparation of hydrogels

**Figure 1.**
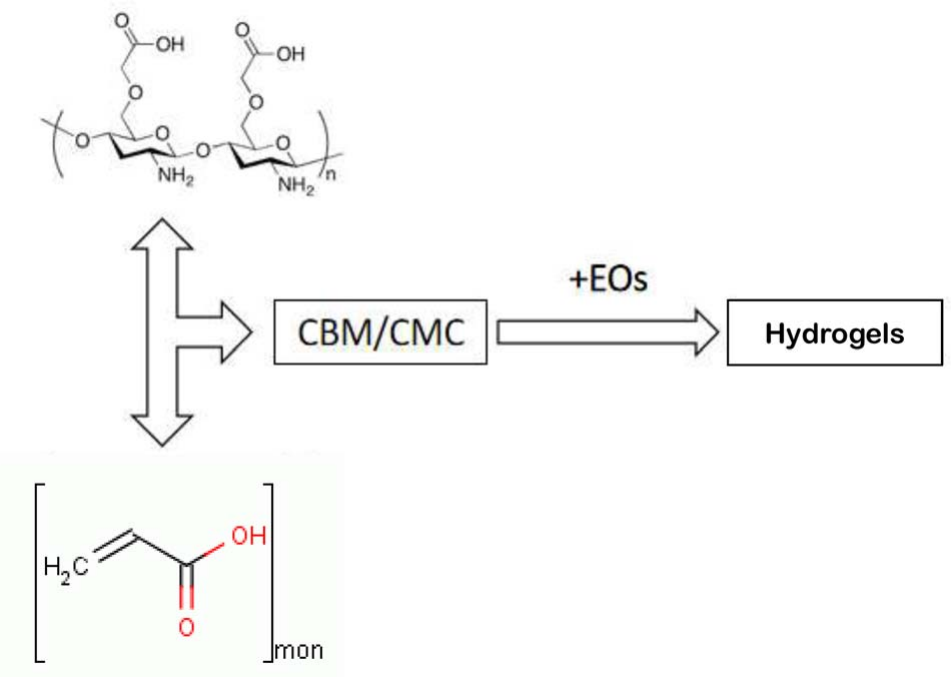
Schematic diagram of antibacterial polysaccharide-based hydrogels preparation. *CBM* carbomer940, *CEO* cumin essential oil, *EO* essential oil

Preparation was carried out by following the previously reported method [15]. In a 200 mL beaker, 0.5% (w/v) CBM was prepared by dissolving 0.5 g of CBM in deionized water, and the resultant solution was kept at ambient temperature overnight. Subsequently, CMC (10% (w/v), 5 mL) was added, and the CBM/CMC hydrogel was stirred for 5 min after adjusting the pH to 7 using triethanolamine. Afterwards, EEO, GEO or CEO (5 mL) was added slowly to the beaker with 50 μL of Tween 20 (CAS:9005-64-5), followed by uniform mixing at 25°C for 5 min. Finally, CBM/CMC/EEO, CBM/CMC/GEO and CBM/CMC/CEO hydrogels were obtained by adding up to 100 g of deionized water to the gel system and stirring the mixture at 25°C for 10 min. A schematic diagram of hydrogel preparation is shown in Figure 1.

### Antimicrobial activity

The antimicrobial activity of the hydrogels was determined against *E. coli* and *S. aureus* bacterial strains by employing turbidimetric analysis [22]. The bacterial strains were incubated in LB broth, with shaking at 150 rpm (37°C) until an absorbance at 600 nm (OD_600_) of 0.5 was achieved. After incubation, the hydrogels or positive control (PC, 50 μL) were mixed with 150 μL of mixed bacterial suspension with a concentration of 10^5^–10^6^ CFU/mL, followed by reaction for 6 h. A 96-well microplate reader was used to measure the OD_600_. Gentamicin sulfate was used as the PC, whereas sterile water was used as the negative control (NC). The antibacterial rate (AR) of the samples for *E. coli* or *S. aureus* was calculated as follows:

(1) \begin{equation*} \mathrm{AR}=\left({\mathrm{OD}}_{\mathrm{NC}}-{\mathrm{OD}}_{\mathrm{sample}}\right)/{\mathrm{OD}}_{\mathrm{NC}}\times 100\% \end{equation*}

where OD denotes the absorbance of the solution at 600 nm, NC denotes the negative control solution and sample denotes the test sample solution. The measurements were repeated thrice, and an average value was reported.

### Hydrogel characterization

#### Fourier-transform infrared spectroscopy

Fourier-transform infrared (FT-IR) spectroscopy of the freeze-dried CBM/CMC, CBM/CMC/EEO, CBM/CMC/GEO and CBM/CMC/CEO hydrogels was carried out by mixing with KBr for pressing the pellets. Attenuated total reflectance (ATR) mode was used on a Thermo (USA) Nicolet iS50 FT-IR Spectrometer. The spectra were collected over the wavenumber range 4000–650 cm^−1^ with a resolution of 4 cm^−1^ and 128 co-added scans.

#### X-Ray diffraction

The X-ray diffraction (XRD) curves of the freeze-dried CBM/CMC, CBM/CMC/EEO, CBM/CMC/GEO and CBM/CMC/CEO hydrogels were recorded using a Bruker D8 ADVANCE (Germany) X-ray powder diffractometer with Cu Kα radiation (λ =0.154 nm), in the diffraction angle range 10–60° at 40 kV and 40 mA.

#### Scanning electron microscopy

The surface morphology of the CBM/CMC, CBM/CMC/EEO, CBM/CMC/GEO and CBM/CMC/CEO hydrogels was analyzed using a scanning electron microscope (JSM-840, Japan) at 10.0 kV accelerating voltage. To avoid structural collapse, the hydrogels were kept at −80°C overnight before freeze-drying for 48 h. The freeze-dried hydrogels were sputtered with a thin layer of gold under vacuum for 20 min to ensure conductivity of the hydrogels.

#### Gas chromatography-mass spectrometry

Gas chromatography-mass spectrometry (GC–MS) analyses of three kinds of essential oils were carried out on an Agilent 7890-5977A GC–MS system (USA) equipped with a HP-5MS capillary column (30 m length × 0.25 mm diameter × 0.25 μm film thickness). The essential oils were diluted with ethyl ether and 0.2 μL was injected in split mode with a split ratio of 1:10. The oven temperature was initially set at 50°C for 1 min, then programmed to 80°C at a rate of 5°C/min and hold for 2 min, then programmed to 180°C at a rate of 10°C/min and hold for 5 min and finally programmed to 220°C at 10°C/min and hold for 2 min. Helium was used as carrier gas at a flow rate of 1.0 mL/min.

For MS determinations, the ionization voltage of the mass spectrometer was 70 eV, with an ion source temperature of 230°C, scan mass range of 40–500 amu and solvent delay time of 2 min. The essential oil compounds were identified by comparison with the NIST 14 spectra library and reported as relative percentages of the total peak area.

### Hydrogel properties

#### Rheological analysis

As viscoelastic materials, hydrogels exhibit unique rheological properties. Oscillatory rheological analysis of the CBM/CMC, CBM/CMC/EEO, CBM/CMC/GEO and CBM/CMC/CEO hydrogels was carried out using an AR2000ex rheometer (TA, UK) in parallel-plate mode (50 mm diameter) at 25°C. The frequency sweep measurement (0.01% strain) was used for gaining information about the storage (G') and loss (G") moduli.

#### Viscosity and swelling rate

The viscosity of the CBM/CMC, CBM/CMC/EEO, CBM/CMC/GEO and CBM/CMC/CEO hydrogels was measured using an NDJ-5S rotary viscometer after placing the hydrogels (100 g) in a beaker.

The original mass (*m*_0_) was calculated by placing the freeze-dried (for 2 d) hydrogels in deionized water at 25°C and storing for 5 min. Subsequently, the hydrogels were weighed after swelling and equilibriating (*m*_a_). The swelling ratio was estimated from the following equation:

(2) \begin{equation*} \mathrm{Swell}\kern0.17em \mathrm{rate}\;\left(\%\right)=\left({\mathrm{m}}_{\mathrm{a}}-{\mathrm{m}}_0\right)/{\mathrm{m}}_0\times 100\% \end{equation*}

#### Water loss rate and water vapor transmission rate

The original mass (*m*_0_) was calculated after placing the hydrogels (1 g) in 2 mL centrifuge tubes. The tubes were subsequently dried in an oven for 18 h at 37°C. The water loss rate was eventually calculated by weighing the tubes once every hour (*m*_a_) during drying as well as at the end (*m*_b_), using the formula:

(3) \begin{equation*} \mathrm{Water}\kern0.17em \mathrm{loss}\kern0.17em \mathrm{rate}\left(\%\right)=\left({\mathrm{m}}_{\mathrm{a}}-{\mathrm{m}}_0\right)/\left({\mathrm{m}}_0-{\mathrm{m}}_{\mathrm{b}}\right)\times 100\% \end{equation*}

Water vapor transmission rate (WVTR) of the CBM/CMC, CBM/CMC/EEO, CBM/CMC/GEO and CBM/CMC/CEO hydrogels was calculated using a gravimetric method, according to the ASTM E96–00 standard. For this, 10 mL of deionized water was added to a glass vial with a diameter of 18 mm, and the mouth of the vial was covered with a layer of gauze. Hydrogel dressing (0.5 g) was used to uniformly cover the gauze, and the mouth of the vial was subsequently sealed. The initial mass (*W*_0_) was accurately weighed. The vial was placed in a silica gel dryer at 37°C and subsequently removed after 12, 24, 36 and 48 h for weighing (*W*_t_). WVTR (g/m^2^/day) was calculated as follows:

(4) \begin{equation*} \mathrm{WVTR}=\left({\mathrm{W}}_0-{\mathrm{W}}_{\mathrm{t}}\right)/{10}^6\mathrm{A}\times \mathrm{T} \end{equation*}

where, *A* is the cross-sectional area of the vial (m^2^) and *T* is the duration for which the hydrogels are placed in the dryer (day). An average of three measurements was taken for each sample.

#### *In vitro* cell culture

L929 cells were cultured in Dulbecco’s Modified Eagle’s Medium (DMEM) with 10% fetal bovine serum (FBS) and antibiotics (100 U/mL penicillin and 100 μg/mL streptomycin) at 37°C overnight in an incubator (Thermo Forma 3111) in an atmosphere containing 5% CO_2_. The adherent cells were digested with 0.25% trypsin and sub-cultured. The L929 cells were inoculated in 96-well plates at a cell density of 1 × 10^5^ per well and were cultured for 9–12 h until the cells adhered to the wall.

#### Indirect cytotoxicity of hydrogels

The indirect cytotoxicity of the CBM/CMC, CBM/CMC/EEO, CBM/CMC/GEO and CBM/CMC/CEO hydrogels was evaluated using the MTT assay [23,24]. Briefly, the freeze-dried hydrogels were sterilized by exposure to the UV light for 2 h. Subsequently, hydrogel extracting liquid solutions were prepared by soaking the hydrogels at different concentrations (1, 10, 100, 1000, 10 000 μg/mL) in DMEM containing serum at 37°C for 24 h. After sterilization with a 0.22 μm filter, the culture medium in the 96-well plates was removed by using fresh DMEM culture medium as well as the extracting liquids corresponding to the CBM/CMC, CBM/CMC/EEO, CBM/CMC/GEO and CBM/CMC/CEO hydrogels for 24 h. Subsequently, 20 μL of 5 mg/mL MTT solution was added to the wells. The solution was further cultured at 37°C for 4 h. Afterwards, the solution was removed from each well and 150 μL of dimethylsulfoxide was added. The absorbance at a wavelength of 490 nm was subsequently measured with a microplate reader (DNM-9602), along with recording the number of each plate. The cell viability (%) of the hydrogels was calculated as:

(5) \begin{eqnarray*} \mathrm{Cell}\kern0.17em \mathrm{viability}\% &&={\mathrm{OD}}_{490}\left(\mathrm{hydrogel}\right)/{\mathrm{OD}}_{490}\left(\mathrm{control}\right)\nonumber\\ &&\quad\times 100\% \end{eqnarray*}

#### Migration assay of L929 cells

The migration of the fibroblast L929 cells was examined by employing the method described by Balekar *et al*. [25]. Briefly, the L929 cells (8 × 10^5^ cells/mL) in DMEM containing FBS (10%) were seeded in each well of a 6-well plate, followed by incubation at 37°C with 5% CO_2_. After a confluent monolayer of the L929 cells was formed, a sterile pipette tip was used to generate a horizontal scratch in each well. Any cellular debris was removed by washing with PBS and adding 1 mL of fresh medium. The cells were treated with PBS and hydrogel extracting liquids at a concentration of 1 μg/mL (PBS served as a negative control). The hydrogel extracting liquid solution concentration based on the performed cytotoxicity assay.

Images of each well were taken using a microphotograph (XDS-500C, Shanghai) at 0, 24 and 48 h. To determine the migration of the L929 cells, the images were analyzed using ImageJ software. For this, after calibration, the uncovered area was delimited with a specific tool to calculate its value. The covered area (%) of the samples was calculated as:

(6) \begin{eqnarray*} \mathrm{Covered}\kern0.17em \mathrm{area}\% &&=\mathrm{covered}\kern0.17em \mathrm{area}\left(24\;\mathrm{or}\;48\mathrm{h}\right)/\nonumber\\ &&\quad\mathrm{covered}\kern0.17em \mathrm{area}\left(0\mathrm{h}\right)\times 100\% \end{eqnarray*}

#### *In vivo* studies

Six-week-old male KM mice (*n* = 84, weight = 28.5 ± 2 g) were purchased from Hunan SJA Laboratory Animal Co. Ltd (Hunan, China). Animal experiments were performed following the National Institute of Health Guide for the Care and Use of Laboratory Animals, China. The procedures for care and use of animals were approved by Experimental Animal and Animal Experiments Center of Shantou University. All reasonable efforts were made to minimize the suffering of the mice.

For the analysis, 84 mice were randomly divided into three groups (28 mice in each group) and acclimatized to the laboratory conditions for ~7 days before wounding. Afterwards, an intraperitoneal injection of 5% chloral hydrate (10/mL) was administered to anesthetize the mice. Before surgery, the dorsal surface hair was removed with 8% (w/v) sodium sulfide, followed by sterilization with 75% alcohol. Subsequently, second-degree burn wounds with a diameter of 1.5 cm were created on the back of each mouse by using a scalding apparatus (YLS-5Q, Yiyan, China) at 100°C with 0.1 kg compression for 10 s. Afterwards, the wounded groups were cured with MEBO Shirun Shaoshang Gao (PC), CBM/CMC/EEO (GEL) and normal saline representing the negative control (NC). Images of the scalds were taken daily, and wound healing rate was calculated using ImageJ software.

For histological analyses, eight mice each were harvested at days 3, 7, 14 and 21, and the wound sites and surrounding skin were excised. After removing the fur in 8% (w/v) sodium sulfide and cleaning with cold saline, the samples were dehydrated in graded ethanol and subsequently embedded in paraffin. The central wound sections with a thickness of 5 μm were fixed on poly-L-lysine-coated glass slides and stained with hematoxylin and eosin (H&E) as well as Masson’s trichrome (Beyotime Institute of Biotechnology, China). Images of the processes of re-epithelialization, granulation tissue formation and collagen deposition of the tissue were acquired with a Nikon ECLIPSE 80i microscope (Nikon, Japan).

For enzyme-linked immunosorbent assay (ELISA) analyses, the wound skin was mixed with normal saline at a ratio of 1:9 and was placed in a tissue homogenizer (OES-Y50, Tiangen, Beijing) for full grinding to achieve a 10% tissue grinding solution. The supernatant of the tissue grinding solution was collected after centrifugation at 3000 rev/min for 20 min, and the concentrations of interleukin- 6 (IL-6), tumor necrosis factor-α (TNF-α), TGF-β1, vascular endothelial growth factor (VEGF) and EGF were determined using an ELISA kit (Solarbio, Beijing, China), following the manufacturer’s protocols. The concentrations were recorded as pg/mL. All calibrations and analyses were repeated in triplicate.

### Statistical analysis

Data were analyzed with GraphPad Prism 7 (GraphPad Software, Inc., La Jolla, CA, USA). Statistical comparisons of hydrogel properties, cell culture data and mouse model were performed with Student’s t-test. Results are presented as mean ± SD. Statistical comparisons between hydrogel groups were performed by analysis of variance/Kruskal–Wallis non-parametric tests. IBM® SPSS® Statistical software (version 22.0) was used for multivariate regression analysis. *P* values ≤0.05 were considered to be significant.

## Results

### Antibacterial activity

The CBM/CMC hydrogel can effectively improve its antibacterial activity with the addition of plant essential oils (especially against gram-negative bacteria). *E. coli* and *S. aureus* were selected to assess the antibacterial properties of the developed hydrogels. As shown in Table 1, the antibacterial efficiency of the CBM/CMC hydrogel was 16.49 ± 1.58% for *S. aureus* and −28.41 ± 0.99% for *E. coli*; the inhibition rates of EEO, GEO and CEO towards *S. Aureus* were 49.06 ± 1.52%, 20.67 ± 2.36% and 16.08 ± 0.99%, respectively, whereas the rates towards *E. coli* were 2.37 ± 0.11%, 2.23 ± 0.08% and 2.96 ± 0.29%, respectively. Therefore, owing to their reliable antibacterial activity, EEO, GEO and CEO were confirmed to be effective antibacterial agents. Although the antibacterial activity significantly increased on incorporating EEO, GEO or CEO into the CBM/CMC hydrogel, the CBM/CMC/EEO hydrogel was observed to possess the highest antibacterial activity towards *S. aureus* (46.26 ± 2.22%) and *E. coli* (63.05 ± 0.99%).

**Table 1:** Antibacterial activity of CBM/CMC, PC, EEO, GEO, CEO, CBM/CMC/PC, CBM/CMC/EEO, CBM/CMC/GEO and CBM/CMC/EEO

**Sample code**	***Escherichia coli***	***Staphylococcus aureus***
CBM/CMC	−28.41 ± 0.99%^a^	16.49 ± 1.58%^a^
PC (gentamicin)	41.04 ± 0.54%^b^	71.28 ± 0.21%^b^
EEO	2.37 ± 0.11%^c^	49.06 ± 1.52%^c^
GEO	2.23 ± 0.08%^c^	20.67 ± 2.36%^d^
CEO	2.96 ± 0.29%^c^	16.08 ± 0.99%^a^
CBM/CMC/PC	0.71 ± 0.14%^d^	16.70 ± 0.24%^a^
CBM/CMC/EEO	46.26 ± 2.22%^e^	63.05 ± 0.99%^e^
CBM/CMC/GEO	18.21 ± 1.20%^f^	38.41 ± 0.21%^f^
CBM/CMC/CEO	22.90 ± 2.15%^g^	53.67 ± 0.42%^g^

*CBM* carbomer 940, *CMC* carboxymethyl chitosan, *EEO* eucalyptus essential oil, *GEO* ginger essential oil, *CEO* cumin essential oil, *PC* positive control. Values are given as mean ± SD (*n* = 3). The concentration of PC used was 50 μg/mL, the concentrations of essential oils used was 50 mg/mL. ^a–g^Different superscript letters in the same column indicate significantly different values (*p* < 0.05)

### Structural characterization of hydrogels

#### Scanning electron microscopy

**Figure 2.**
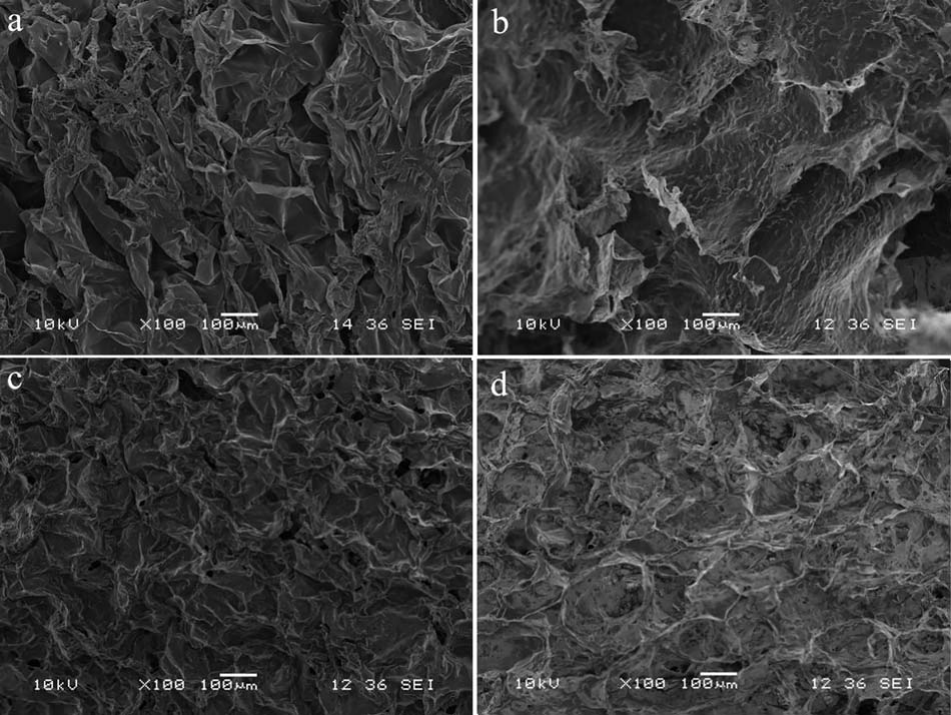
SEM images of the (**a**) CBM/CMC, (**b**) CBM/CMC/EEO, (**c**) CBM/CMC/GEO and (**d**) CBM/CMC/CEO hydrogels. *CBM* Carbomer 940, *CMC* Carboxymethyl chitosan, *EEO* Eucalyptus essential oil, *GEO* ginger essential oil, *CEO* cumin essential oil, *SEM* scanning electron microscope

Scanning electron microscopy (SEM) was used to characterize the CBM/CMC, CBM/CMC/EEO, CBM/CMC/GEO and CBM/CMC/CEO hydrogels. Figure 2a–d presents images of the hydrogels, exhibiting a lamellar network structure. The surface morphology of the CBM/CMC hydrogel (Figure 2a) was observed to have a smooth and homogeneous skeleton with an aperture ratio of 28.40 ± 1.21%, while small bumps on the skeleton of the hydrogel network structure were observed in the other hydrogels with smaller aperture ratios of 6.55 ± 0.79%, 16.30 ± 1.07% and 13.04 ± 0.89% (Figure 2b–d, respectively) due to the introduction of essential oils. Moreover, the laminar network structure of the CBM/CMC/EEO hydrogel (Figure 2b) displayed larger pores than the CBM/CMC/GEO (Figure 2c) and CBM/CMC/CEO (Figure 2d) hydrogels.

#### FT-IR and XRD

**Figure 3.**
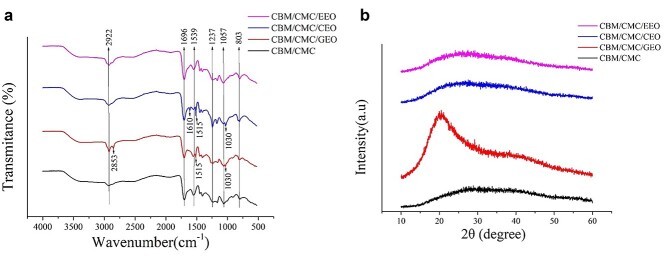
The characteristics of morphosis parameter of CBM/CMC (black line), CBM/CMC/GEO (red line), CBM/CMC/CEO (blue line) and CBM/CMC/EEO (pink line) hydrogels. (**a**) FT-IR spectra of the four kinds of hydrogels. (**b**) XRD patterns of the four kinds of hydrogels. *CBM* carbomer 940, *CMC* carboxymethyl chitosan, *EEO* eucalyptus essential oil, *GEO* ginger essential oil, *CEO* cumin essential oil, *XRD* X-ray diffraction

FT-IR was used to confirm the non-covalent interactions between CBM, CMC and the essential oils. The FT-IR spectra of the CBM/CMC, CBM/CMC/EEO, CBM/CMC/GEO and CBM/CMC/CEO hydrogels are illustrated in Figure 3a. The spectra of CBM/CMC, CBM/CMC/EEO, CBM/CMC/GEO and CBM/CMC/CEO hydrogels exhibited minor differences. The CBM/CMC hydrogels showed characteristic peaks at 2922, 1696, 1539, 1237, 1057 and 803 cm^−1^. Hydrogen bonding and hydrophobic interaction were the main non-covalent bonds between the essential oil and CBM/CMC. The CBM/CMC/EEO hydrogel exhibited similar peaks to the CBM/CMC hydrogel, though the intensity of the peaks at 2922 and 1696 cm^−1^ was slightly enhanced. Compared with the CBM/CMC hydrogel, the CBM/CMC/GEO hydrogel exhibited additional peaks at 2853 and 1515 cm^−1^. Comparing CBM/CMC and CBM/CMC/CEO, CBM/CMC/CEO was observed to exhibit additional peaks at 1661 and 1512 cm^−1^.

In addition, GC–MS was used to analyze the main components of the essential oils, as displayed in the online supplementary material. Based on the peak area, the most active components of EEO, GEO and CEO were observed to be eucalyptol, 1,3-cyclohexadiene and anethole, respectively (Figures S3 and Tables S1–S3, see online supplementary material).

Figure 3b shows the XRD patterns of the CBM/CMC, CBM/CMC/GEO, CBM/CMC/CEO and CBM/CMC/EEO hydrogels. A high-intensity, broad diffraction peak at 2θ = 25.1° was observed in the CBM/CMC, CBM/CMC/EEO and CBM/CMC/CEO hydrogels. In addition, the CBM/CMC/GEO hydrogel exhibited a crystalline phase with a sharp and narrow peak at 2θ = 20.2°.

### Properties of hydrogels

#### Rheological analysis

**Figure 4.**
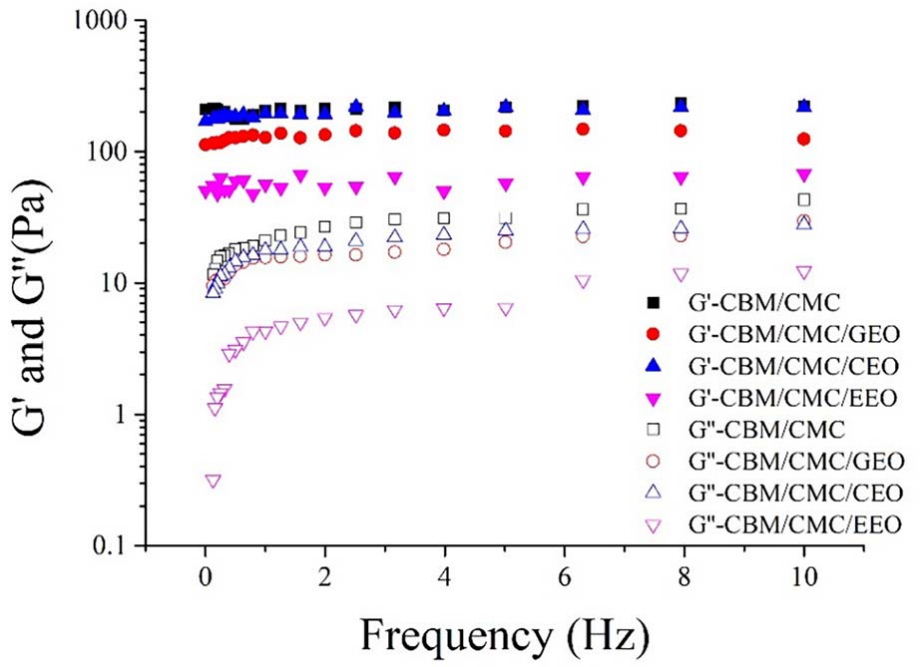
Storage (G′) and loss (G″) moduli of the CBM/CMC, CBM/CMC/GEO, CBM/CMC/CEO and CBM/CMC/EEO hydrogels as a function of angular frequency. *CBM* carbomer 940, *CMC* carboxymethyl chitosan, *EEO* eucalyptus essential oil, *GEO* ginger essential oil, *CEO* cumin essential oil

Oscillatory shear rheology was employed to investigate the rheological properties of the hydrogels, and the storage (G′, elastic component) and loss (G″, viscous component) moduli of the hydrogels were measured as a function of either strain or frequency. The rheological profiles of the hydrogels in the frequency range 0.1–10 Hz are summarized in Figure 4. The G′ values of all hydrogels were observed to be much higher than the G″ values. Moreover, the G′ and G″ values of the CBM/CMC/EEO, CBM/CMC/GEO and CBM/CMC/CEO hydrogels were lower than the CBM/CMC hydrogel due to the introduction of the plant essential oils (Tables S4–S5, see online supplementary material).

#### Viscosity, swelling, water loss and water vapor transmission of the hydrogels

**Figure 5.**
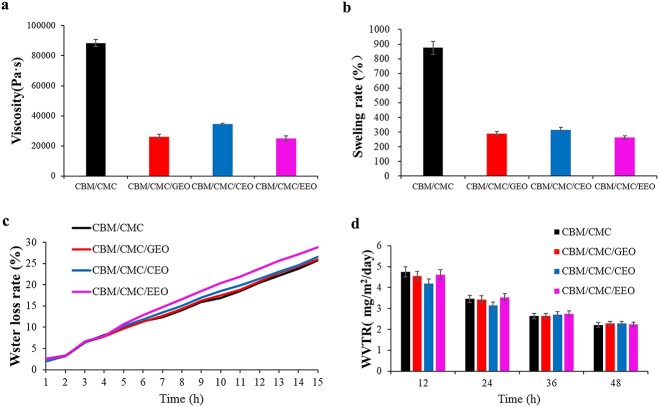
Various physical parameters of CBM/CMC (black line), CBM/CMC/GEO (red line), CBM/CMC/CEO (blue line) and CBM/CMC/EEO (pink line) hydrogels. (**a**) Viscosity of the four kinds of hydrogels. (**b**) Swelling rate of the four kinds of hydrogels. (**c**) Water loss rate of the four kinds of hydrogels. (**d**) Water vapor transmission rate (WVTR) rate of the four kinds of hydrogels. *CBM* carbomer 940, *CMC* carboxymethyl chitosan, *EEO* eucalyptus essential oil, *GEO* ginger essential oil, *CEO* cumin essential oil

An effective hydrogel for wound repair should possess appropriate viscosity (no sticking to the wound so as to avoid secondary damage), specific swelling performance (equilibrium absorption of the wound drainage), superior water retention and optimal water vapor transmission performance (to satisfy the gas-exchange needs of cells). In this respect, Figure 5 shows the viscosity (a), swelling (b), water loss rate (c) and WVTR (d) of the CBM/CMC, CBM/CMC/GEO, CBM/CMC/CEO and CBM/CMC/EEO hydrogels, respectively.

From high to low, the viscosity values of the hydrogels were 88 433, 34 533, 26 133 and 24 966 Pa s^−1^ for CBM/CMC, CBM/CMC/CEO, CBM/CMC/GEO and CBM/CMC/EEO, respectively (Figure 5a). As shown in Figure 5b, the swelling rate of the CBM/CMC, CBM/CMC/GEO, CBM/CMC/CEO and CBM/CMC/EEO hydrogels was determined to be 874.69%, 288.63%, 316.06% and 262.04%, respectively. Thus, the swelling rate exhibited a reduction after incorporation of EEO, GEO or CEO. The water loss rate and WVTR of the developed hydrogels were measured next. The CBM/CMC/EEO hydrogel exhibited higher water loss rate and WVTR as compared to the other hydrogels. The water loss rate of the CBM/CMC/EEO hydrogel was 30% in 15 h whereas its WVTR was observed to be 2.23 g/m^2^/day (Figure 5c, d).

Overall, in this study, the CBM/CMC/EEO hydrogel exhibited optimal viscosity, swelling rate, water loss rate and WVTR values of 24 966 Pa s^−1^, 262.04%, 25% and 2.23 g/m^2^/day, respectively, which were close to the requirements needed to maintain a suitable fluid balance on the wound bed, facilitated cellular migration and enhanced re-epithelialization. Thus, it was confirmed that the CBM/CMC/EEO hydrogel was the most suitable dressing material for the treatment of injured skin.

#### *In vitro* cell culture

Cell viability and migration are the crucial indicators of biocompatibility [26]. For this reason, the biocompatibility of the developed hydrogels was investigated for L929 cells, the main cell type involved in wound healing.

#### Indirect cytotoxicity

**Figure 6.**
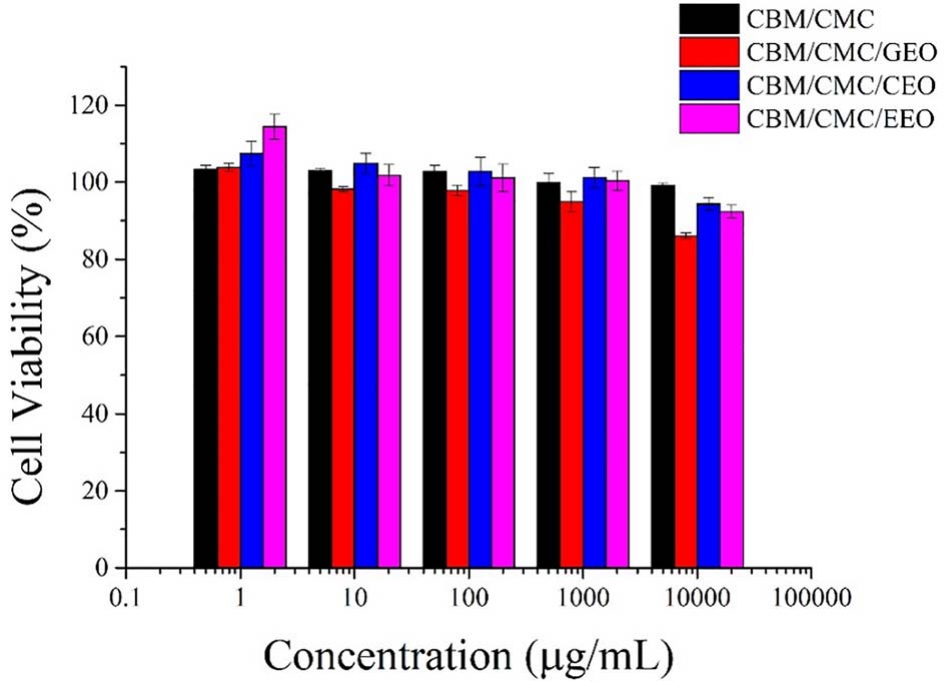
Cell viability of the L929 cells treated with different concentrations of the hydrogel extracts after 24 h. *CBM* carbomer 940, *CMC* carboxymethyl chitosan, *EEO* eucalyptus essential oil, *GEO* ginger essential oil, *CEO* cumin essential oil

The indirect cytotoxicity of the CBM/CMC, CBM/CMC/EEO, CBM/CMC/GEO and CBM/CMC/CEO hydrogels was evaluated by the MTT assay using L929 cells. It was observed from the MTT assay (Figure 6) that the hydrogels did not exhibit any obvious cytotoxicity up to an extraction concentration of 1000 mg/mL. When the hydrogel concentrations increased to 10,000 mg/mL, the CBM/CMC, CBM/CMC/EEO, CBM/CMC/GEO and CBM/CMC/CEO hydrogels revealed cell viability of 99.16%, 92.37%, 94.27% and 86.10%, respectively. Furthermore, the rate of cell viability was observed to decrease slightly as the concentration of the gel extract was increased.

#### Migration results of L929 cells

**Figure 7.**
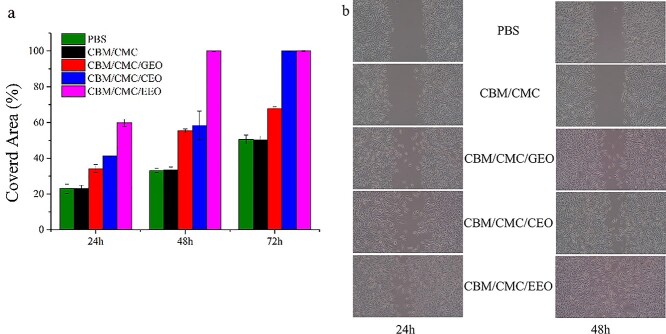
*In vitro* cell experiments of the various hydrogels. (**a**) The percentage of the covered area after treatment with different hydrogel extracts. (**b**) The images of the L929 cell migration after treatment. *CBM* carbomer 940, *CMC* carboxymethyl chitosan, *EEO* eucalyptus essential oil, *GEO* ginger essential oil, *CEO* cumin essential oil

As demonstrated in Figure 7, the CBM/CMC/EEO hydrogel induced significant migration of L929 cells, reaching 59.83% covered area after 24 h and 100.00% after 48 h, however, the other hydrogels could not achieve such performance. Figure 7b presents images representative of the data in Figure 7a. Migration of the L929 cells was attributed to the presence of the essential oil in the composite CBM/CMC/EEO hydrogel, which promoted cell migration [27].

Thus, based on the observed findings, the CBM/CMC/EEO hydrogel exhibited a minimal effect on apoptosis and could promote migration of L929 cells to some extent, which indirectly indicated its superior biocompatibility.

#### *In vivo* studies

The experimental findings obtained so far demonstrated the superior performance of the CBM/CMC/EEO hydrogel among the developed hydrogels, thus the CBM/CMC/EEO hydrogel was chosen as the experimental subject for *in vivo* studies.

#### Full-thickness wound healing efficiency

**Figure 8.**
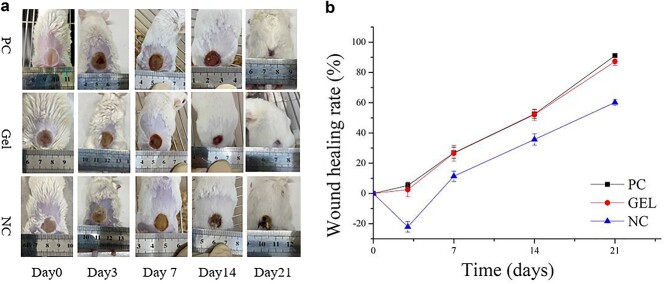
*In vivo* animal experiments with positive drug, CBM/CMC/EEO hydrogel and saline on days 3, 7, 14 and 21 respectively. (**a**) Representative visual wound appearance (the scale presents the dimensions in mm). (**b**) Wound healing rate comparison for treatment. GEL indicated CBM/CMC/EEO hydrogel. *CBM* carbomer 940, *CMC* carboxymethyl chitosan, *EEO* eucalyptus essential oil, *PC* positive control, *NC* negative control

Wound healing comprises a series of complex biological events involving hemostasis, inflammatory response, structure repair and wound systole [26]. In Figure 8a, at day 0, the wounds of the mice were noted to be porcelain white and shriveled. No significant difference was observed in wound morphology among the three groups. On day 3, the GEL (CBM/CMC/EEO) and PC groups exhibited wound contraction without exudate, while the NC group demonstrated diffusion of the wound area. All mice demonstrated burn area contraction and scab formation after 7 days, with the GEL group exhibiting the largest contraction, smooth wound surface and wound healing rate of 22%. At days 14 and 21, all mice were observed to recover further, with the evolution of new skin area and hair. The mice in the GEL and PC groups were almost completely healed, with vigorous growth of new hair, whereas the wounds of the mice in the NC group were still scabbed at day 21. Figure 8b shows the specific wound healing rate of the mice. Overall, the observed results indicated that the GEL group exhibited the most effective healing of burn wounds in mice.

#### Histology of the wound healing site in mice

**Figure 9.**
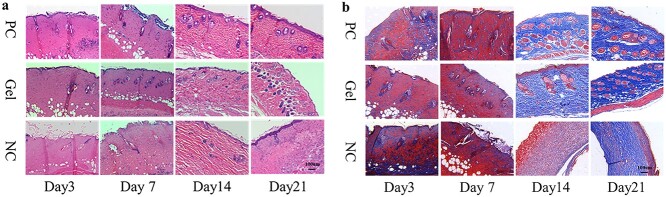
Histology of the wound healing site in the mice with positive drug, CBM/CMC/EEO hydrogel and saline on days 3, 7, 14 and 21 respectively (**a**) The H&E-stained images of the mice wound tissue for treatment. (**b**) Masson-stained images of the mice wound tissue for treatment. GEL indicated CBM/CMC/EEO hydrogel. *CBM* carbomer 940, *CMC* carboxymethyl chitosan, *EEO* eucalyptus essential oil, *PC* positive control, *NC* negative control

Wound healing is generally divided into the following overlapping phases: hemostasis, inflammation, migration, migration and remodeling. To observe the histological changes in the different phases, H&E and Masson’s trichrome staining were employed at predetermined intervals after wound creation (Figure 9).

On days 3 and 7, the groups displayed varying degrees of acute inflammatory reaction, with numerous inflammatory cells (such as macrophages) migrating to the wound site. Histology analysis of the PC and GEL groups revealed the disappearance of the epidermal layer, dermis necrosis, degradation of collagen and reduction/disappearance of the skin accessory organs (such as hair follicles, sweat glands, etc.). On the other hand, the NC group revealed the destruction of the dermis cell structure and severe infiltration of inflammatory cells. At days 14 and 21, the wound defect area was observed to be covered with new epithelium. Compared with the NC group, collagen fibers proliferated in abundance leading to organized collagen deposition in the PC and GEL groups. The number of skin accessory organs in the PC and GEL groups was also significantly enhanced, with epidermis significantly restored with the formation of cuticle.

#### ELISA analysis of the wound healing site

**Figure 10.**
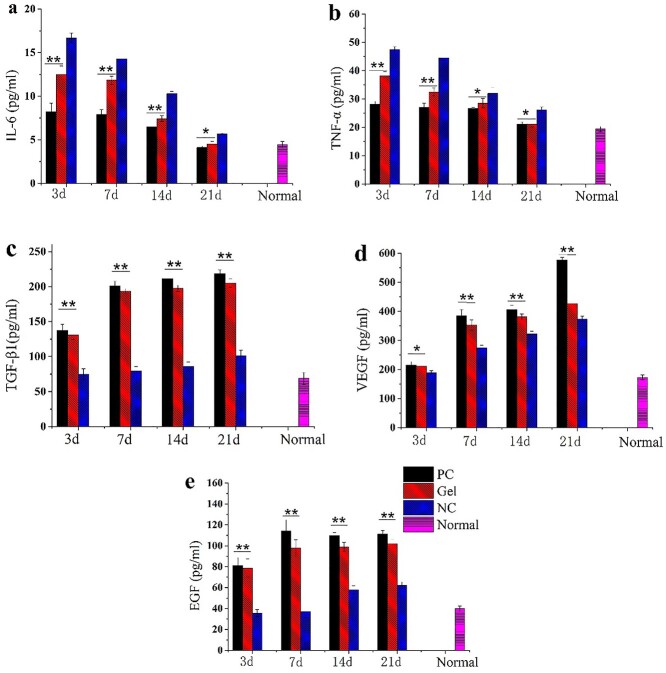
Quantitative analysis of various cytokine content in the wound tissue by employing the ELISA method. The values have been expressed as mean ± SD. One-way ANOVA was used to evaluate the differences. ^*^p < 0.05 and ^**^p < 0.01 *vs* NC (**a**) Quantitative determination of IL-6 content. (**b**) Quantitative determination of TNF-α content. (**c**) Quantitative determination of TGF-β1 content. (**d**) Quantitative determination of VEGF content. (**e**) Quantitative determination of EGF content. GEL indicated CBM/CMC/EEO hydrogel. *PC* positive control, *NC* negative control, *VEGF* vascular epidermal growth factor, *EGF* epidermal growth factor

Inflammatory and growth factors are the potent signaling molecules that act in a coordinated manner in physiological processes such as tissue healing or angiogenesis. In Figure 10a, b, the levels of IL-6 and TNF-α in the mice tissue were noted to decrease with time after generation of the burn wound. The expression of these factors in the GEL group was significantly lower than that in the NC group. As shown in Figure 10c–e, the expression of these factors in the GEL group rose significantly as compared to the NC group. In summary, this study demonstrated that the CBM/CMC/EEO hydrogel (GEL) promoted the reduction of inflammatory factors (IL-6 and TNF-α) towards normal levels as well as the upregulation of growth factors (TGF-β, VEGF and EGF) with time.

## Discussion

In this article, antibacterial hydrogels with essential oils were synthesized, characterized and tested *in vivo* and *in vitro*. During this process, CBM/CMC/EEO hydrogel presents the most promising option as a dressing for wound healing.

Plant essential oils with antibacterial effect could be incorporated to optimize the antibacterial activity of the CBM/CMC hydrogel. In this study *E. coli* was more resistant to the CBM/CMC hydrogel, which is consistent with a previous report suggesting that CMC exhibited poor antibacterial efficiency towards gram-negative bacteria due to the thick cell walls [28]. In addition, the observed antibacterial activity was attributed to the addition of CMC with amidogen [29,30] and the barrier established by CBM [31]. The reports have shown that the addition of essential oils to a matrix contributes toward reducing the required doses of the essential oils, while retaining their antioxidant-antimicrobial activity [32]. We hypothesized that the synergistic effects between essential oils and CBM/CMC enhanced the overall antimicrobial activity against *E. coli* and *S. aureus*, as the network structure of the CBM/CMC hydrogel delays the release of essential oils [33]. Plant essential oils, produced by plants as secondary metabolites, have been reported to possess effective bioactivity and biocompatibility owing to constituents such as terpenoids, phenolics and polyphenols [9,34,35]. In our previous studies, the antibacterial activity of grape seed, rose, bergamot, lemon, chamomile, lavender, tea tree, ginger, cumin and eucalyptus essential oils have been studied. Among these, EEO, GEO and CEO have been reported to exhibit the highest antibacterial activity. Thus, they were chosen as the antibacterial additive for the CBM/CMC hydrogel in this study. In addition, as these essential oils are volatile substances, sustained release of essential oils is inevitable in the process of interaction with a hydrogel matrix. However, we hypothesized that sustained release of essential oil in hydrogels could provide better wound healing effects.

The added EEO, GEO or CEO phase was observed to spread on the surface of the hydrogel network structure, thus making the hydrogel surface rough and promoting the adhesion of cells, making it conducive to wound repair [9,36], which was consistent with the literature studies [30,32]. The large pore size and fast water vapor diffusion of hydrogels signify high application value in the field of wound repair [37]. Therefore, the CBM/CMC/EEO hydrogel, with a porous 3D network and EEO incorporation leading to a rough surface, was confirmed to be an optimal candidate for cell attachment, especially fibroblasts and keratinocytes, for effective wound healing.

The CBM/CMC/EEO, CBM/CMC/GEO and CBM/CMC/CEO hydrogels showed a different infrared spectrum than CBM/CMC hydrogel due to the presence of essential oils. In the FT-IR spectra of the CBM/CMC hydrogel, the absorption peaks at 1696, 1539 and 1237 cm^−1^ corresponded to the stretching vibration of C=O from the carboxylate group, N-H bending vibration and C=N stretching, respectively [15,38].

Based on the XRD results, it was confirmed that the hydrogels exhibited a crystalline or amorphous structure, and the addition of the essential oils did not affect the crystal structure of the CBM/CMC hydrogel, except for GEO, owing to the optimal adaptability between the polymer matrix and essential oils. The CBM/CMC/GEO hydrogel exhibited a crystalline phase with a sharp and narrow peak at 2θ = 20.2° due to the 1,3-cyclohexadiene in GEO [39].

The properties of hydrogels are known to influence cell function and differentiation, thus impacting practical wound repair application [40–44]. Rheological analysis showed the elastic solid-like nature of the hydrogels because the G′ values were observed to be much higher than the G″ values [15,45]. Moreover, EEO, GEO or CEO addition to the CBM/CMC hydrogel would lead to ideal gelation occurring in the CBM/CMC/EEO, CBM/CMC/GEO or CBM/CMC/CEO hydrogel at lower G′ and G″ values. This can be explained by the fact that the addition of plant essential oils changes the network structure of the hydrogels and reduces hydrogel flexibility. As for other properties, the swelling rate of a hydrogel dressing is an important parameter as far as wound repair is concerned. It reflects the ability of the hydrogel to absorb wound exudates. In the present study, the swelling rate exhibited a reduction after incorporation of essential oils, therefore we speculated that the addition of essential oils introduced many hydrophobic groups, which enhanced the hydrophobicity of the hydrogels and made it difficult for water molecules to enter through hydrogen bonding, thus leading to a decrease in the swelling rate of hydrogels. Hence, the CBM/CMC/EEO hydrogel, with a swelling rate of 262.04%, was identified as an ideal candidate hydrogel for effective absorption of wound exudates during wound repair.

Collagen is a main component of the skin and plays an important role in tissue healing by providing tissue strength and an extracellular matrix framework for cell adhesion and migration [46]. The *in vivo* results illustrated that the CBM/CMC/EEO hydrogel treatment induced the regeneration of collagen fibers, epidermis and appendages. On the other hand, the promotion of wound healing during the CBM/CMC/EEO hydrogel treatment might have been due to the 3D net structure of the hydrogel with active ingredients, which helped cell adherence by providing a suitable environment for wounds. This confirmed that the CBM/CMC/EEO hydrogel demonstrated a valid skin-repairing effect.

IL-6 and TNF-α cytokines play an important role in the inflammatory response by emerging in abundance after body injury [47,48]. TGF-β, VEGF and EGF cytokines are related to cell growth and differentiation, formation of granulation and anti-inflammatory hemostasia [49,50]. In the skin wound healing process, platelets release TGF-β and other chemokines, attracting inflammatory cells into the wound and beginning the skin repair process. Then, macrophages promote the release of anti-inflammatory IL-6 by releasing TNF-α, activating macrophage differentiation for the anti-inflammatory phenotype; at the same time, EGF in the wound also promotes the anti-inflammatory differentiation of macrophages. Subsequently, the activated macrophages, fibroblasts and keratinocytes release a lot of VEGF and vascular endothelial cells with new granulation tissue formation, so as to further complete the wound repair process. Due to their important roles in skin tissue repair, these cytokines including IL-6, TNF-α, TGF-β, VEGF and EGF cytokines were selected as the main indicators owing to their important role in skin tissue repair in this study.

## Conclusions

In this study, CBM/CMC/EEO hydrogel was successfully prepared and applied as a burn wound dressing. The essential oil hydrogel demonstrated porous structure, high antibacterial activity, favorable swelling and optimal rheological properties. Moreover, the essential oil hydrogel exhibited superior water retention and water vapor transmission performance. In addition, evaluation of the migration of L929 cells revealed that the CBM/CMC/EEO hydrogel promoted effective cell recovery in 48 h. The CBM/CMC/EEO hydrogel considerably improved burn wound healing in a mouse model. The wound tissue area measurement, histology staining and analysis of IL-6, TNF-α, TGF-β, VEGF and EGF levels collectively demonstrated that the CBM/CMC/EEO hydrogel treatment significantly accelerated burn wound repair in a mouse model. Overall, the developed CBM/CMC/EEO hydrogel presents significant potential as a new type of burn wound dressing.

## Abbreviations

CBM:, Carbomer940; CEO:; Cumin essential oil; CMC:, Carboxymethyl chitosan; EEO:, Eucalyptus essential oil; EGF:, Epidermal growth factor; ELISA:, Enzyme-linked immunosorbent assay; FT-IR:, Fourier-transform infrared; GC:, Gas chromatography; GEO:, Ginger essential oil; H&E: hematoxylin and eosin; IL-6:, Interleukin- 6; MS:, Mass spectrometry; MTT:, 3-(4,5-Dimethyl-2-thiazolyl)-2,5-diphenyl-2-H-tetrazolium bromide; NC:, Negative control; PC:, Positive control; SEM:, Scanning electron microscopy; TGF-β1:, Transforming growth factor-β1; TNF-α:, Tumor necrosis factor-α; VEGF:, Vascular endothelial growth factor; WVTR:, Water vapor transmission rate; XRD:, X-ray diffraction

## Availability of data

The data and material to support the structure of the CBM/CMC/EEO hydrogel, including the molecular weight of the polysaccharide composition, the degree of substitution in CMC, the various essential oils compositions and the oscillatory rheological analysis of CBM/CMC/EEO hydrogel.

## Authors’ contributions

YL conceived and supervised the work; HW made substantial contributions to the interpretation of data for the work; KC and BZ made contributions to the experimental data for the work; ST and WL made contributions to the analysis of data for the work; WZ made contributions to the acquisition of data for the work. All data were generated in-house, and no paper mill was used. All authors agree to be accountable for all aspects of the work ensuring integrity and accuracy.

## Conflict of interest

None declared.

## Supplementary Material

Supplementary_material_tkab041
