# Supplementary material for: Antibacterial polysaccharide-based hydrogel dressing containing plant essential oil for burn wound healing
Source: Burns Trauma. 2021 Dec 22;9:tkab041. doi: 10.1093/burnst/tkab041 (PMC8693078; doi:10.1093/burnst/tkab041)
Supplement: Supplementary_material_tkab041 [file supplementary_material_tkab041.docx]

**S1.** **Determination of molecular weight by gel permeation chromatography (GPC)**

The molecular weight of CMC was determined through gel permeation chromatography (GPC) using an GPC system that was equipped with a TSK-GEL GMPWXL (a series column model No. 008025, Tosoh, Tokyo, Japan) and a differential refraction detector (Shimadzu, Japan). 20 μL CMC solution (2 mg/mL) was injected into the GPC system. The column was eluted with 0.1N NaN3 and 0.06%NaN3 at a flow rate of 0.6 mL/min. Pullulan standards (642 kDa, 334 kDa, 49.4 kDa, 22 kDa, 6.3 kDa) for GPC were used, and a calibration curve was drawn in Figure S-1.

**Figure S-1 Calibration curve of pullulan standards**

CMC exhibited two main peaks in the GPC chromatograms as shown in Figure S-2. On the basis of the equation derived from the standard curve, the molecular weights of CMC were calculated by differentiation and the molecular weight distribution curve were shown in Figure S-3. In conclusion, CMC in this study is mainly concentrated in 195.7kDa and 2.0kDa.

**
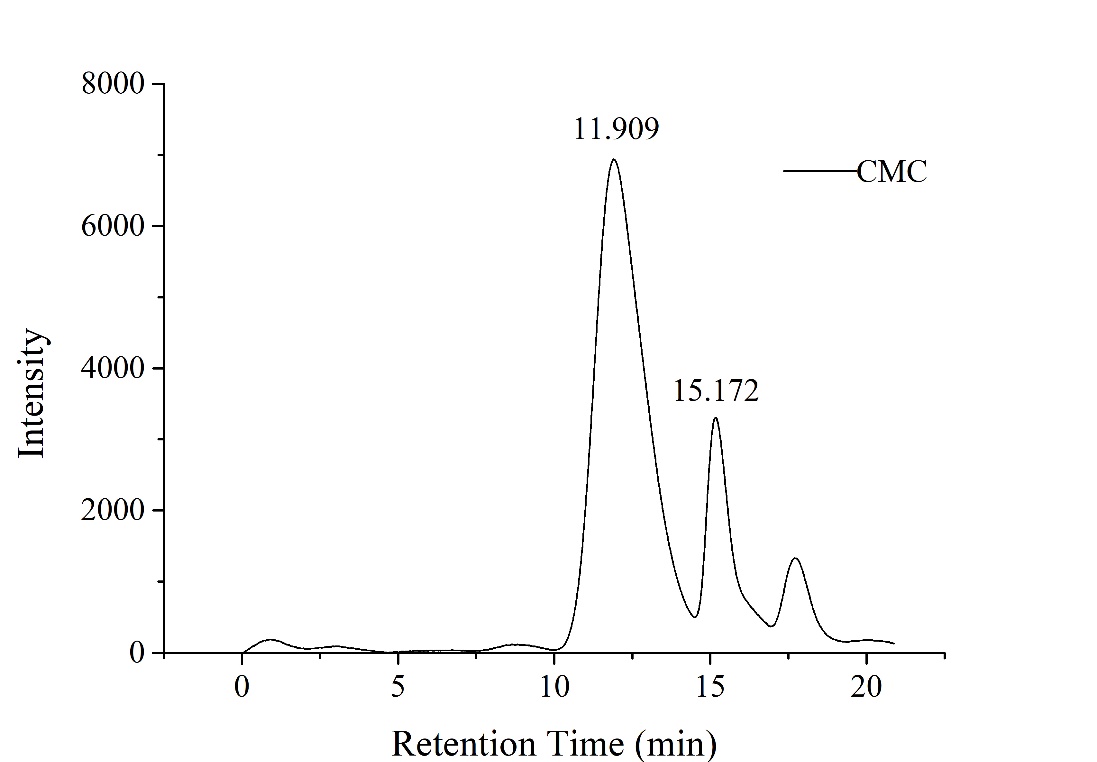
**

**Figure S-2 GPC elution of CMC**

**
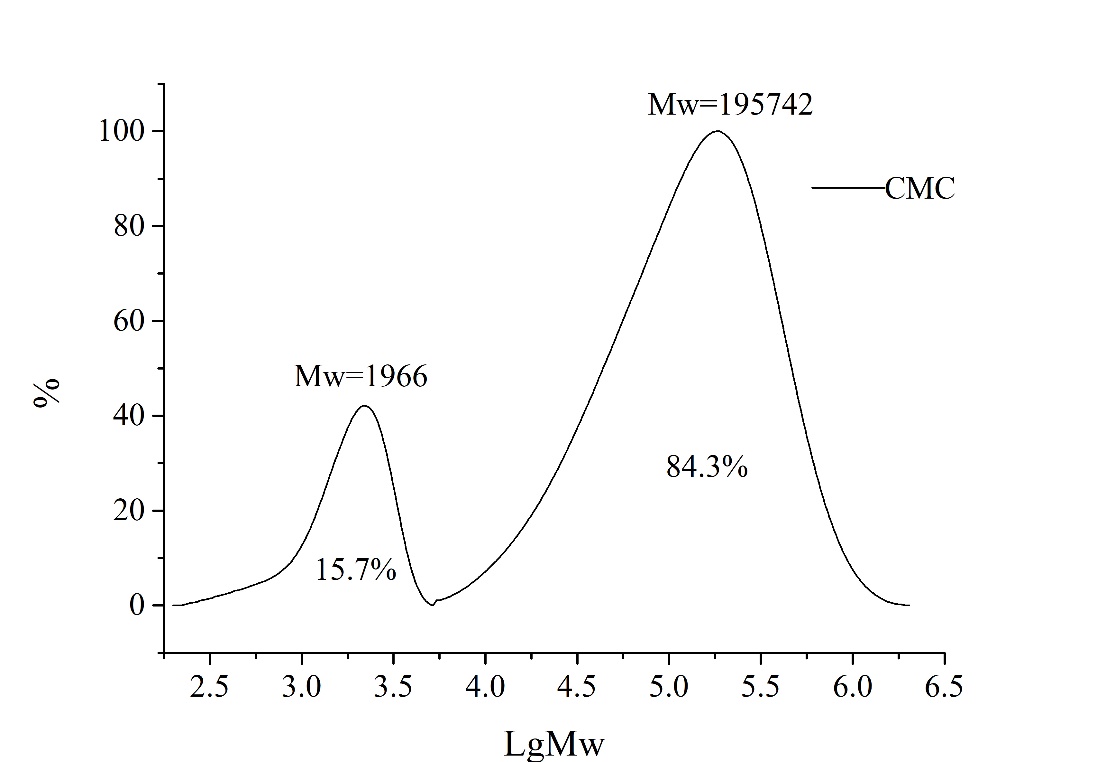
**

**Figure S-3 Molecular weight distribution curve of CMC**

**S2.** **Determination of degree of substitution (DS) in CMC by potentiometric titration method**

0.2 g CMC were weighed into 50 mL standard HCl (0.1M) solutions, the CMC was completely dissolved with a magnetic stirrer for about 30min. Then, titrated with standard NaOH (0.1 M) solution were titrated slowly and the pH was recorded by pH meter. According to the two inflection points of the titration curve, noted the consumption NaOH volume V_1_ and V_2_(mL), respectively. The formula are as follows:

DS=0.203A/(1-0.058A)

A= (V_2_ – V_1_)×C/W

In conclusion, the DS of CMC was calculated as 73.73% according to potential titration curve in Figure S-4.

**Figure S-4 Potential titration curve of CMC**

**S3.** **Composition analysis of essential oils by Gas Chromatography-Mass Spectrometer (GC-MS)**

GC-MS analyses of ginger essential oil (GEO), cumin essential oil (CEO) and eucalyptus essential oil (EEO) were carried out on an Agilent 7890-5977A GC-MS system (USA) equipped with HP-5MS capillary column (30m length × 0.25 mm diameter × 0.25 μm film thickness). The essential oils were diluted with ethyl ether and 0.2 μL was injected in the split mode with split ratio 1:10. The oven temperature was initially set at 50 ℃ for 1 min, then programmed to 80 ℃ at a rate of 5 ℃/min and hold for 2 min, then programmed to 180 ℃ at a rate of 10 ℃/min and hold for 5 min, finally programmed to 220 ℃ at 10 ℃/min and hold for 2 min. Helium was used as carrier gas at a flow rate 1.0 mL/min.


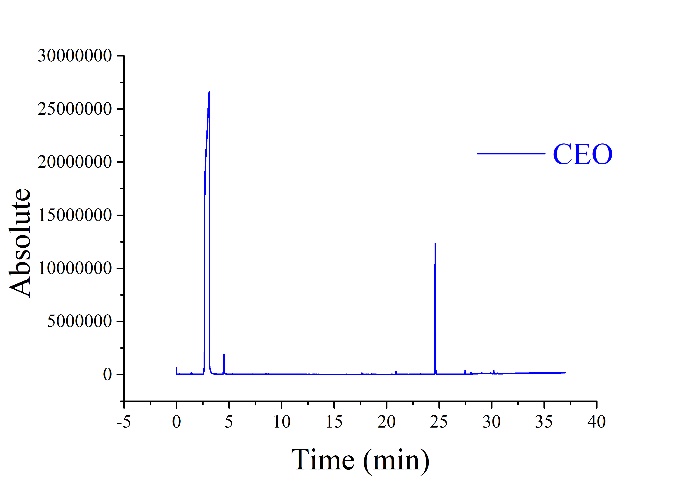

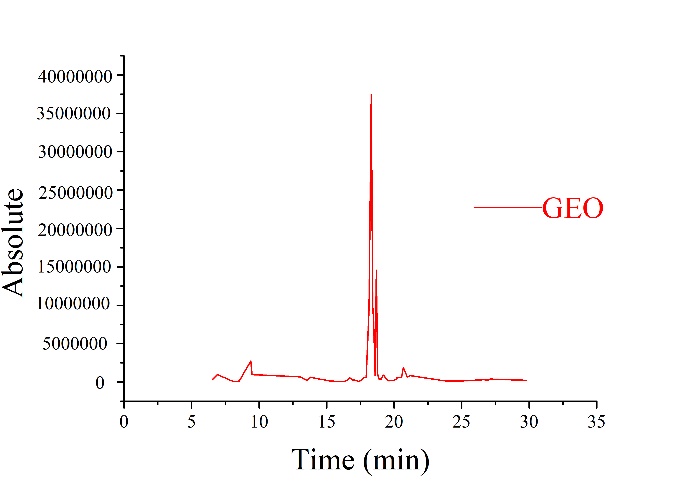
For Mass spectrometer determination, ionization voltage of mass spectrometer was 70 eV, with ion source temperature of 230 ℃ and scan mass range of 40-500 amu, solvent delay time of 2 min. The compounds of essential oils were identified by comparison with of NIST 14 spectra library and reported as relative percentages of the total peak area, which were shown in Figure S-5 and Table S-1, S-2, and S-3.


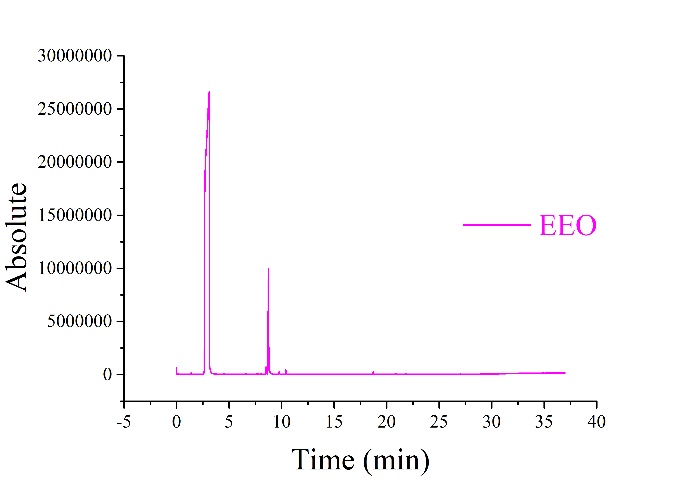


**Figure S-5 Total ion chromatography diagram of three kind of essential oils: the representative samples from top to bottom are ginger essential oil (GEO), cumin essential oil (CEO) and eucalyptus essential oil (EEO), respectively.**

**Table S-1** Main compounds of the GEO with relative percentage more than 0.1%

| **(GEO)** | | | |
| --- | --- | --- | --- |
| Peak Number | Retention time/min | Compound | Peak Area (%) |
| 1 | 6.538 | hept-2-ene | 0.32 |
| 2 | 6.916 | Camphene | 1.12 |
| 3 | 9.409 | β-Phellandrene | 2.14 |
| 4 | 9.485 | Eucalyptol | 1.01 |
| 5 | 13.033 | endo-Borneol | 0.65 |
| 6 | 16.574 | 1,2,4-Metheno-1H-indene | 0.34 |
| 7 | 16.705 | α-Copaene | 0.61 |
| 8 | 16.922 | Cyclohexane | 0.3 |
| 9 | 17.745 | cis-β-Farnesene | 0.68 |
| 10 | 17.936 | Alloaromadendrene | 0.39 |
| 11 | 18.143 | Benzene | 7.18 |
| 12 | 18.196 | Germacrene | 2.42 |
| 13 | 18.305 | 1,3-Cyclohexadiene | 39.81 |
| 14 | 18.379 | Methanoazulene | 3.1 |
| 15 | 18.426 | γ-Muurolene | 7.15 |
| 16 | 18.474 | β-Bisabolene | 9.81 |
| 17 | 18.598 | Germacrene | 1.28 |
| 18 | 18.683 | Cyclohexene | 14.92 |
| 19 | 19.195 | 1,5-Cyclodecadiene | 0.99 |
| 20 | 20.706 | 2-Butanone | 0.65 |

**Table S-2** Main compounds of the CEO with relative percentage more than 0.1%

| **(CEO)** | | | |
| --- | --- | --- | --- |
| Peak Number | Retention time/min | Compound | Peak Area (%) |
| 1 | 1.51 | Hexane | 0.07 |
| 2 | 4.505 | α-Pinene | 15.48 |
| 3 | 8.51 | D-Limonene | 0.63 |
| 4 | 17.635 | Linalool | 1.00 |
| 5 | 20.9 | Estragole | 2.61 |
| 6 | 24.635 | Anethole | 74.53 |
| 7 | 27.48 | Benzaldehyde | 2.06 |
| 8 | 28.04 | 3-Methyl-4-nitrobenzyl alcohol | 0.99 |
| 9 | 29.045 | 2-Propanone, 1-(4-methoxyphenyl) | 0.46 |
| 10 | 29.9 | 2-Hydroxymandelic acid | 0.50 |
| 11 | 30.195 | 3-Methyl-2-butenoxy | 1.38 |
| 12 | 30.555 | 3-Hydroxymandelic acid | 0.27 |

**Table S-3** Main compounds of the EEO with relative percentage more than 0.1%

| **(EEO)** | | | |
| --- | --- | --- | --- |
| Peak Number | Retention time/min | Compound | Peak Area (%) |
| 1 | 6.59 | Bicyclo(3.1.0)-hexane, 4-methy | 0.50 |
| 2 | 7.66 | α-Phellandrene | 0.31 |
| 3 | 7.745 | β-Myrcene | 0.58 |
| 4 | 8.03 | 4-Carene | 0.54 |
| 5 | 8.51 | D-Limonene | 5.82 |
| 6 | 8.74 | Eucalyptol | 83.27 |
| 7 | 9.765 | γ-Terpinene | 1.85 |
| 8 | 10.39 | o-Cymene | 3.46 |
| 9 | 18.72 | Caryophyllene | 2.08 |
| 10 | 20.85 | Humulene | 0.88 |
| 11 | 21.815 | α-Terpineol | 0.70 |

**S4. The hydrogels values of** **G' and G''**

The oscillatory rheological analysis of the CBM/CMC, CBM/CMC/EEO, CBM/CMC/GEO and CBM/CMC/CEO hydrogels was carried out using AR2000ex rheometer (TA, UK) in the parallel plate mode (50 mm diameter) at 25 °C. The frequency sweep measurement (0.01% strain) was used for gaining information about the storage (G') and loss (G") moduli, which were shown in Table S-4 and S-5, respectively.

**Table S-4** The hydrogels values of G'

| **G'** | | | | |
| --- | --- | --- | --- | --- |
| HZ | CBM/CMC | CBM/CMC/GEO | CBM/CMC/CEO | CBM/CMC/EEO |
|  |  |  |  |  |
| 10 | 222.3 | 124.7 | 217.1 | 67.8 |
| 7.943 | 233.8 | 144.1 | 218.1 | 64.31 |
| 6.31 | 223.7 | 148.1 | 207.8 | 64.32 |
| 5.012 | 217.6 | 143.3 | 218.6 | 57.52 |
| 3.981 | 206.6 | 145.7 | 205.1 | 50.02 |
| 3.162 | 218.1 | 139 | 197.4 | 64.59 |
| 2.512 | 211.1 | 144.5 | 219.4 | 54.26 |
| 1.995 | 213 | 134.2 | 193.2 | 53.45 |
| 1.585 | 204.6 | 127.6 | 192.7 | 67.01 |
| 1.259 | 213.1 | 138.3 | 194.8 | 53.07 |
| 1 | 204.8 | 128.3 | 194.4 | 56.37 |
| 0.7943 | 192.1 | 133.7 | 182.9 | 47.64 |
| 0.631 | 178.7 | 131.1 | 192.9 | 60.8 |
| 0.5012 | 177.2 | 127.1 | 185.5 | 59.8 |
| 0.3981 | 185.8 | 127.7 | 184.5 | 51.47 |
| 0.3162 | 200.6 | 121.9 | 188.8 | 51.15 |
| 0.2512 | 202.3 | 117.5 | 182.6 | 62.93 |
| 0.1995 | 209.2 | 117.6 | 178.9 | 47.91 |
| 0.1585 | 213.2 | 115.8 | 177.4 | 54.27 |
| 0.1259 | 214.2 | 116.2 | 179 | 54.7 |
| 0 | 210.6 | 112.9 | 170.4 | 50.55 |

**Table S-5** The hydrogels values of G''

| **G''** | | | | |
| --- | --- | --- | --- | --- |
| HZ | CBM/CMC | CBM/CMC/GEO | CBM/CMC/CEO | CBM/CMC/EEO |
|  |  |  |  |  |
| 10 | 43.07 | 29.59 | 27.73 | 12.33 |
| 7.943 | 36.6 | 22.81 | 25.94 | 11.77 |
| 6.31 | 36.31 | 22.66 | 25.61 | 10.5 |
| 5.012 | 31.18 | 20.39 | 25.03 | 6.496 |
| 3.981 | 30.99 | 18.05 | 23.2 | 6.445 |
| 3.162 | 30.38 | 17.13 | 22.17 | 6.248 |
| 2.512 | 28.87 | 16.34 | 20.91 | 5.716 |
| 1.995 | 26.73 | 16.32 | 18.9 | 5.402 |
| 1.585 | 24.15 | 16.02 | 18.74 | 5.004 |
| 1.259 | 22.93 | 15.77 | 17.9 | 4.648 |
| 1 | 21.11 | 15.65 | 17.77 | 4.272 |
| 0.7943 | 19.21 | 15.43 | 16.3 | 4.231 |
| 0.631 | 18.33 | 14.39 | 15.56 | 3.565 |
| 0.5012 | 18.07 | 13.72 | 14.59 | 3.115 |
| 0.3981 | 16.83 | 12.09 | 12.99 | 2.904 |
| 0.3162 | 16.08 | 10.78 | 11.55 | 1.573 |
| 0.2512 | 15.88 | 10.73 | 11.21 | 1.467 |
| 0.1995 | 14.75 | 10.6 | 9.954 | 1.361 |
| 0.1585 | 12.8 | 10.17 | 9.058 | 1.125 |
| 0.1259 | 11.62 | 9.482 | 8.295 | 0.3177 |
